# Fisheye Image Detection of Trees Using Improved YOLOX for Tree Height Estimation

**DOI:** 10.3390/s22103636

**Published:** 2022-05-10

**Authors:** Jiayin Song, Yue Zhao, Wenlong Song, Hongwei Zhou, Di Zhu, Qiqi Huang, Yiming Fan, Chao Lu

**Affiliations:** Department of Mechanical and Electrical Engineering, Northeast Forestry University, Harbin 150040, China; songjy@nefu.edu.cn (J.S.); zhaoyue@nefu.edu.cn (Y.Z.); wlsong139@126.com (W.S.); zd1998@nefu.edu.cn (D.Z.); h_qiqi@nefu.edu.cn (Q.H.); fanyiming@nefu.edu.cn (Y.F.); luhanyu@nefu.edu.cn (C.L.)

**Keywords:** tree height estimation, equidistant projection, deep learning, fisheye image

## Abstract

Tree height is an essential indicator in forestry research. This indicator is difficult to measure directly, as well as wind disturbance adds to the measurement difficulty. Therefore, tree height measurement has always been an issue that experts and scholars strive to improve. We propose a tree height measurement method based on tree fisheye images to improve the accuracy of tree height measurements. Our aim is to extract tree height extreme points in fisheye images by proposing an improved lightweight target detection network YOLOX-tiny. We added CBAM attention mechanism, transfer learning, and data enhancement methods to improve the recall rate, F_1_ score, AP, and other indicators of YOLOX-tiny. This study improves the detection performance of YOLOX-tiny. The use of deep learning can improve measurement efficiency while ensuring measurement accuracy and stability. The results showed that the highest relative error of tree measurements was 4.06% and the average relative error was 1.62%. The analysis showed that the method performed better at all stages than in previous studies.

## 1. Introduction

Tree height is one of the most critical parameters in quantitative forest observation. Tree height research has important implications for urban road planning, air pollution control, and carbon neutrality. In large-scale forest stock and biomass estimation, tree height can estimate forest stock and biomass [1,2]. However, the characteristics of trees and the complex environment make direct measurements difficult. In addition, wind disturbance also increases the difficulty of measurements.

Traditional forest surveys mostly use theodolites for measurements. Theodolites can accurately obtain forest parameter factors. However, theodolites are time- and manpower-consuming. The measurement of theodolites has long survey cycles and low efficiency, so the real-time and spatial integrity of the data is difficult to keep consistent. A commonly used tool for measuring tree height is the ultrasonic rangefinder. It has the advantage of portability and real-time access to data. However, it is subject to human factors and wind speed and varies significantly from measurement to measurement. Therefore, the measurement of standing tree height remains a problem that researchers are working to improve.

Some researchers use airborne laser scanning (ALS) and terrestrial laser scanning (TLS) to measure tree heights [3,4,5]. However, both ALS and TLS have certain drawbacks, such as ALS is generally expensive and TLS is inconvenient to carry. Measurements using drone equipment are more costly and have poor endurance. Kędra et al. compared single-image photogrammetry (SIP) and terrestrial laser scanning (TLS). The results show that, compared to TLS, SIP can successfully apply tree-like structure feature extractions in mature forests [6]. Digital image-based measurement methods have obvious advantages in terms of economic considerations. Photogrammetry has come a long way with the development of photography and computer vision, which has led researchers to look for new ways to measure tree heights [7,8].

The monocular vision measurement of ordinary cameras has the advantages of easy image acquisition and fewer calculation parameters required for calibration. However, normal cameras have small viewing angles and require long shooting distances when measuring large-scene objects. In 2000, Zhang proposed a tessellation grid calibration method based on pinhole cameras [9]. Before this, camera calibration often required high precision calibrators while Zhang’s calibration method only required a printed checkerboard grid. After acquiring images of different directions from the checkerboard calibration plate, correspondence between the target in the 3D space and the image points on the 2D image plane can be established. After that, the internal and external parameters of the camera can be solved. However, the method is only applicable to ordinary pinhole cameras and the calibration effect is not suitable for wide-angle cameras. Scaramuzza proposed an omnidirectional camera modeling method based on the Taylor series model, which focuses on the calibration of fisheye lenses and refractive lenses within 195° [10]. The omnidirectional camera calibration method is widely used in fisheye camera calibrations because of its simple, accurate, and easy-to-use features.

Photogrammetry uses vision-based measurement methods to identify measured objects in an image. Then, it uses image processing technology to obtain coordinates of the central part of the image. The obtained coordinates are brought into the corresponding mathematical model and the measured value of the measured object can be calculated [11,12,13]. The extraction of extreme points in the central part of the image adopts a clustering algorithm. However, uncompressed images consume much memory which results in a long execution time for the clustering algorithm [14,15]. The image quality will be degraded after compression, which will affect measurement accuracy [16]. Researchers need to manually set the number of clusters in the clustering process based on experience [17]. In 2016, Redmon et al. first proposed the YOLO algorithm [18]. After that, the YOLO series of algorithms were widely used in agriculture, medicine, and intelligent transportation [19,20,21]. The YOLO series of algorithms have shown superior performance. With the continuous development of image detection algorithms, the accuracy based on deep learning has continued to improve. In 2021, Bochkovskiy and other researchers proposed YOLOv4, whose accuracy has been significantly improved compared with previous detection algorithms [22]. After YOLOv4, the YOLOX object detection network appeared and showed superior performance [23]. The YOLO series algorithm can accurately extract image feature points after training, as well as it consumes less time and does not require human experience intervention [24,25,26,27].

In this study, we propose a highly robust method for the non-contact measurements of tree height. The method uses a smartphone with a fisheye lens to capture images. The improved YOLOX algorithm is used for tree recognition and image coordinate extraction, improving recognition accuracy and efficiency.

## 2. Materials and Methods

### 2.1. Establishment of the Measurement Model

All characters and abbreviations appearing in this paper are located in Table A1 in the Appendix A. The parameters of the fisheye lens and smartphone are in Table A2 in the Appendix A. The measurement system model of this method is constructed based on the principle of the equidistant projection model. Here, $P(x_{w},y_{w},z_{w})$ is the target point in the world coordinate system and $P^{\prime }$ is the imaging point corresponding to P in the camera coordinate system.

According to the isometric projection theorem, the projection relationship is expressed as follows:

(1) $$r^{\prime }=fw$$

(2) $$w=\tan^{-1}(r/L)=\tan^{-1}[{\left(x_{w}{}^{2}+y_{w}{}^{2}\right)}^{1/2}/L]$$

where $r^{\prime }$ is the distance from the point $P^{\prime }$ to the optical axis, f is the object square focal length of the optical system, w is the incident angle of the point P relative to the optical axis, and L is the horizontal distance between the point in the world coordinate system and the center of the fisheye lens. Due to the distortion of the fisheye lens, to ensure the uniformity of the image, the distortion coefficient λ is introduced to obtain the following:

(3) $$r^{\prime }=\lambda fw$$

The camera plane center point is $O_{c}(x_{0},y_{0})$, the coordinates of the $P^{\prime }$ point are $\left(x_{c},y_{c}\right)$, and the coordinates of the P point are $\left(x_{w},y_{w},z_{w}\right)$. If the distortion coefficient components of $x_{c}$ and $y_{c}$ axes are $\lambda_{x}$ and $\lambda_{y}$, then:

(4) $$\left\{ \begin{array}{l} x_{c}-x_{0}=r^{\prime }\cos\theta =\lambda_{x}fw\cos\theta \\ y_{c}-y_{0}=r^{\prime }\sin\theta =\lambda_{y}fw\sin\theta \end{array}\right.$$

(5) $$\left\{ \begin{array}{l} \cos\theta =x_{w}/{\left(x_{w}{}^{2}+y_{w}{}^{2}\right)}^{1/2}\\ \sin\theta =y_{w}/{\left(x_{w}{}^{2}+y_{w}{}^{2}\right)}^{1/2} \end{array}\right.$$

where θ is the azimuth of point P and also the azimuth of point $P^{\prime }$ in the camera coordinate system. The coordinates of the center point o in the image pixel coordinate system are $\left(u_{0},v_{0}\right)$; $P^{\prime }$ is obtained by equidistant projection $P^{\prime }$ and the relationship between the camera coordinate system and the corresponding points in the image pixel coordinate system is as follows:

(6) $$\left\{ \begin{array}{l} u-u_{0}=m_{x}(x_{c}-x_{0})=\lambda_{x}m_{x}f(x_{c}-x_{0})\\ v-v_{0}=m_{y}(y_{c}-y_{0})=\lambda_{x}m_{y}f(y_{c}-y_{0}) \end{array}\right.$$

where, $m_{x}$ and $m_{y}$ are the amplification factors. $k_{x}=\lambda_{x}m_{x}f,k_{y}=\lambda_{y}m_{y}f$.

From Equations (1)–(6), the relationship between image coordinates and world coordinates is as follows:

(7) $$\left\{ \begin{array}{l} u=\frac{x_{w}k_{x}}{\sqrt{x_{w}{}^{2}+y_{w}{}^{2}}}\tan^{-1}\frac{\sqrt{x_{w}{}^{2}+y_{w}{}^{2}}}{L}+u_{0}\\ v=\frac{y_{w}k_{y}}{\sqrt{x_{w}{}^{2}+y_{w}{}^{2}}}\tan^{-1}\frac{\sqrt{x_{w}{}^{2}+y_{w}{}^{2}}}{L}+v_{0} \end{array}\right.$$

**Figure 1:**
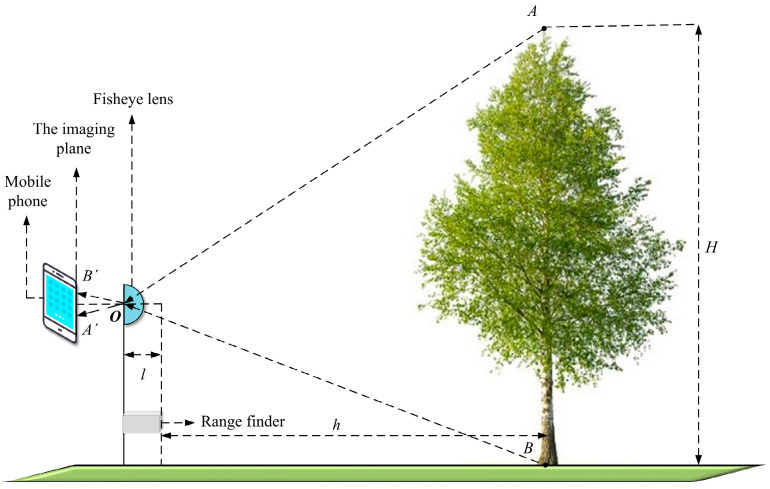
Measurement system model.

The measurement system model consists of a fisheye lens, a rangefinder, and a smartphone. The measurement system model is shown in Figure 1.

When using a smartphone equipped with a fisheye lens to take a picture of a single tree, $A^{\prime }\left(u_{A^{\prime }},v_{A^{\prime }}\right)$ and $B^{\prime }\left(u_{B^{\prime }},v_{B^{\prime }}\right)$ are the corresponding points in the image coordinate system, which are also the extreme points of the tree. The relationship between the corresponding points in the world coordinate system and the image pixel coordinate system are as follows:

(8) $$\left\{ \begin{array}{l} x_{w}=\frac{L}{\sqrt{1+{\left[\frac{k_{x}(v-v_{0})}{k_{y}(u-u_{0})}\right]}^{2}}}\tan\frac{(u-u_{0})\sqrt{1+{\left[\frac{k_{x}(v-v_{0})}{k_{y}(u-u_{0})}\right]}^{2}}}{k_{x}}\\ y_{w}=\frac{k_{x}(v-v_{0})}{k_{y}(u-u_{0})}x \end{array}\right.$$

The coordinates of the center point of the image coordinate system are $o(u_{0},v_{0})$. $k_{x}$ and $k_{y}$ are the distortion coefficients of the fisheye image, which can be obtained by the camera calibration method. L is the horizontal distance, which the following formula can obtain:

(9) L=h+l

where h is the horizontal distance in the world coordinate system and l is the virtual imaging distance of the fisheye lens. Through the transformation relationship between coordinate systems, the following formula can be obtained:

(10) $$\left\{ \begin{array}{l} x_{w}=\frac{L}{\sqrt{1+{\left[\frac{k_{x}(v-v_{0})}{k_{y}(u-u_{0})}\right]}^{2}}}\tan\frac{(u-u_{0})\sqrt{1+{\left[\frac{k_{x}(v-v_{0})}{k_{y}(u-u_{0})}\right]}^{2}}}{k_{x}}\\ y_{w}=\frac{k_{x}(v-v_{0})L}{k_{y}(u-u_{0})\sqrt{1+{\left[\frac{k_{x}(v-v_{0})}{k_{y}(u-u_{0})}\right]}^{2}}}\tan\frac{(u-u_{0})\sqrt{1+{\left[\frac{k_{x}(v-v_{0})}{k_{y}(u-u_{0})}\right]}^{2}}}{k_{x}} \end{array}\right.$$

According to Equation (10), H is the result obtained by the measurement system model [28].

(11) $$H={\left[{\left(x_{A}-x_{B}\right)}^{2}+{\left(y_{A}-y_{B}\right)}^{2}\right]}^{1/2}$$

In Equation (11), H is the final calculated tree height value; the extreme points $A^{\prime }\left(u_{A^{\prime }},v_{A^{\prime }}\right)$ and $B^{\prime }\left(u_{B^{\prime }},v_{B^{\prime }}\right)$ of the tree are the parameters needed to calculate the tree height.

**Figure 2:**
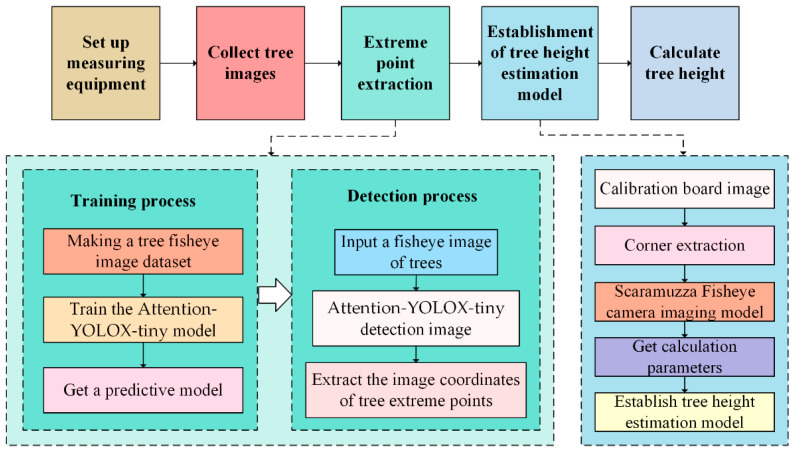
Tree height calculation process.

Tree extrema are defined as the highest and lowest points of a tree. The improved YOLOX-tiny object detection network can detect the complete tree and extract tree extreme points. After that, the extracted extreme points are brought into the tree height estimation model to calculate the tree’s height. The general flow chart for estimating tree height is shown in Figure 2.

The procedure for calculating tree height is as follows:

- Set up measuring equipment. A smartphone with a fisheye lens is required to set up the measuring equipment.
- Acquire images. After training is complete, only one image of the tree under test needs to be collected.
- Extract extreme points. Deep learning methods can perform this step quickly and accurately.
- Build a tree height calculation model. This step only needs to be done once during the initial calculation.
- Calculate tree height. Obtain results and perform error analysis.

### 2.2. Improved Target Detection Network

YOLOX is similar to the previous YOLO version. The whole YOLOX can be divided into the following parts: CSPDarknet is the backbone feature extraction network of YOLOX. The input image is extracted in CSPDarknet and the extracted features are the feature layer, which is the feature set of the input image. FPN is an enhanced feature extraction network of YOLOX. The feature extraction module is performed using the obtained effective feature layers. YOLOX not only upsamples the fused features but also downsamples the fused features. YOLO Head is a classifier of YOLOX with three enhanced effective feature layers obtained by CSPDarknet and FPN. Each feature layer has a width, height, and number of channels. YOLOX uses the Focus network structure, which is used in YOLOV5. In a picture, every other pixel takes a value to get four independent feature layers and then these four separate feature layers are stacked. First, the input image is subjected to shallow feature extraction. Then, the three feature layers are outputted to the feature fusion part for deep feature extraction.

The attention mechanism refers to the panorama of the image that the human vision can focus on a certain local area. The attention mechanism is also used in the research of deep learning. The idea is to use new weights to highlight key points in the image data and train the network. The model identifies the location of the target of interest in the dataset. CBAM (Convolutional Block Attention Module) is a lightweight attention module [29].

CBAM first learns the weight distribution from the relevant features. Then, it feeds the weights back to the features to enhance the network feature recognition ability. The convolutional layer plays a crucial role in the process of feature extraction. The number of channels in each convolution layer is only related to the number of convolution kernels. The feature map is the result of the convolution operation of the input image. However, the convolution layer contains many convolution kernels and the generated feature map will also have a corresponding number of channels. The existence of the attention model plays a role in channel filtering.

**Figure 3:**
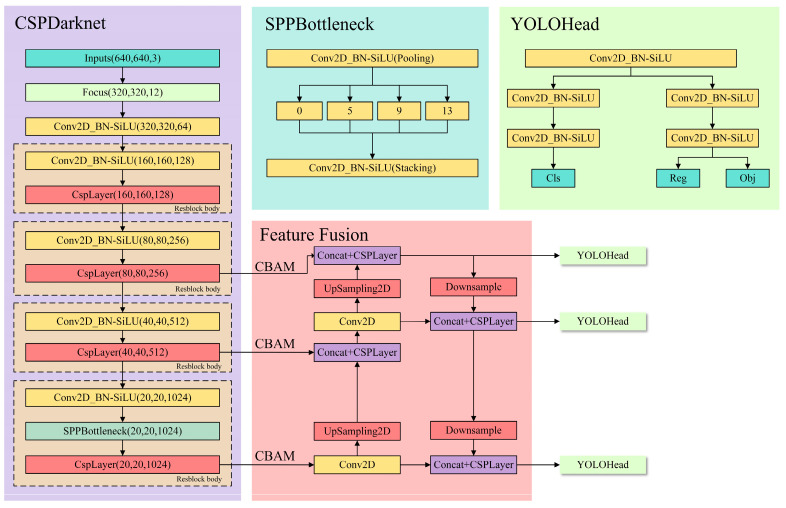
Attention-YOLOX-tiny network structure.

In this study, embedding the CBAM module keeps the original YOLOX-tiny structure (Attention-YOLOX-tiny). The network structure of Attention-YOLOX-tiny is shown in Figure 3.

**Figure 4:**
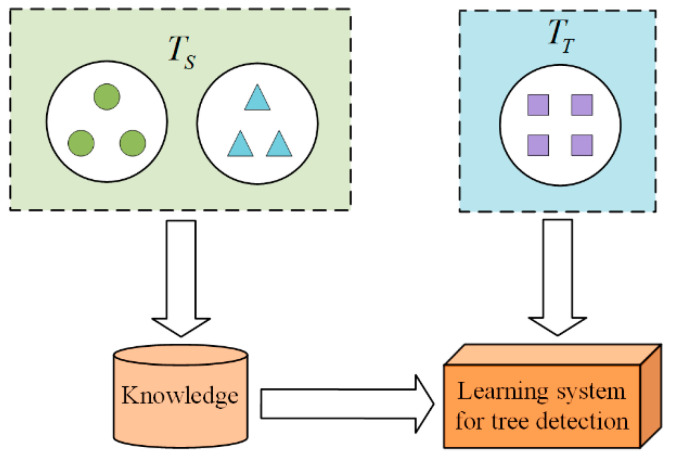
Transfer learning.

To obtain better detection results, the transfer learning method is used to load the pre-trained model [30]. The learning model needs to learn related source tasks on the source domain and then transfer the knowledge to the target task on the target domain to improve the model’s performance on specific tasks. Given the source domain ($D_{S}$) and the source task ($T_{S}$), the target domain ($D_{T}$) and target task ($T_{T}$), the knowledge acquired, and $T_{S}$ help the model solve the prediction function ($f_{T}$) of $T_{T}$ on $D_{T}$. The transfer learning process is shown in Figure 4.

**Figure 5:**
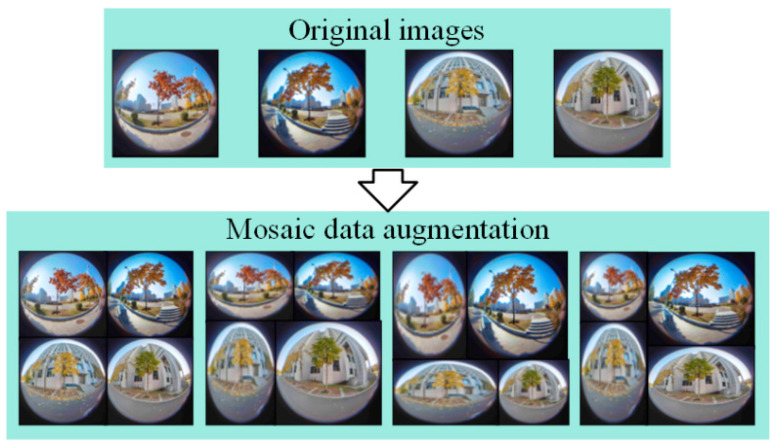
Mosaic data augmentation.

During the training process, mosaic data augmentation is used to augment the dataset. Mosaic data enhancement refers to reading four pictures at a time, flipping, scaling, and changing the color gamut of the four pictures, respectively. Then, it positions according to the positions of the four directions and combines the pictures and frames. The mosaic data enhancement method is shown in Figure 5.

## 3. Experimental Results and Analysis

### 3.1. Validation of Fisheye Lens Measurement Model

After obtaining the required parameters of the tree height measurement model, the tree height measurement model is used to measure the distance between the corner points of the black and white chessboard. The measurement model is verified by comparing the actual distance between the corner points.

**Figure 6:**
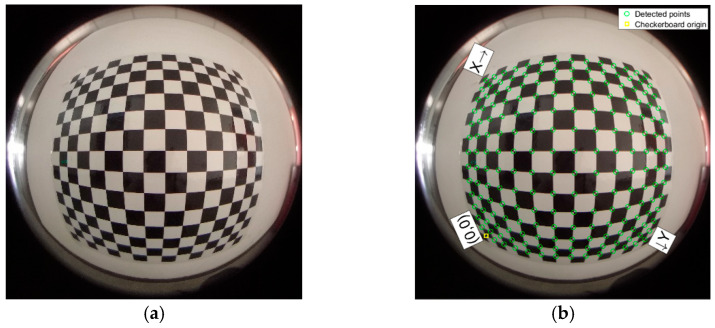
Checkerboard and corner extraction. (**a**) Fisheye image; (**b**) Corner extraction.

The optical centroid is found using the Scaramuzza model and the fisheye image is processed by directly calling the matlab2018b fisheye lens calibration toolbox. The extraction of the checkerboard and checkerboard corners is shown in Figure 6.

**Figure 7:**
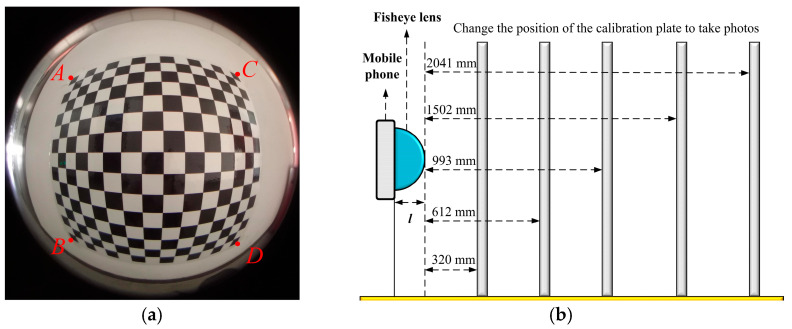
Corner point position selection and calibration plate position selection. (**a**) Corner point position, *A*, *B*, *C*, and *D* are corner points; (**b**) Five positions of the calibration plate.

The following steps were performed: camera calibration, calculation of the distortion coefficient corresponding to all corners in each chessboard, and the average value was taken for subsequent calculations. Five sets of chessboard diagrams with different distances were taken to verify the accuracy of the measurement model, as shown in Figure 7.

Three sets of distances, *AB*, *AC*, and *AD*, were taken on the chessboard, the error was analyzed, and the accuracy of the measurement model was verified. Table 1 shows the calculation results of the distance between *AB*, *AC*, and *AD*.

The analysis and calculation results show that the average relative error is 0.823%. The measurement error of this measurement model is low and can be applied to the measurement of tree height.

### 3.2. Tree Detection and Extreme Point Extraction

**Figure 8:**
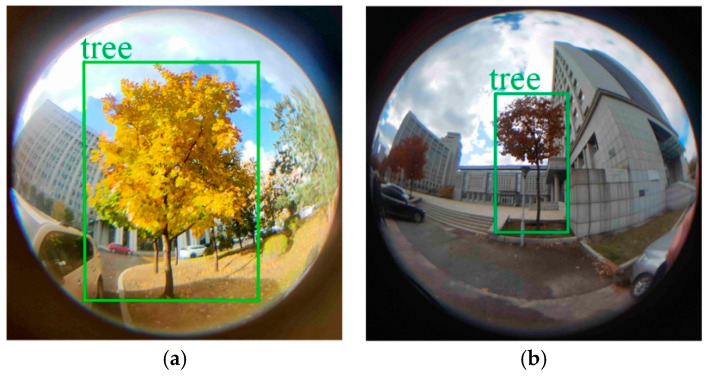
Fisheye image annotation under different weather conditions. (**a**) Sunny images annotation; (**b**) Cloudy images annotation.

Before taking pictures, 178 randomly selected trees from the Northeast Forestry University were marked. To improve the robustness of the model and fully consider the effect of light in the experiments, a smartphone equipped with a fisheye lens was used to capture fisheye images on sunny and cloudy days. We acquired 1035 photos (including 537 on sunny days and 498 on cloudy days). The image acquisition time covers the whole day. The dataset contains different light intensities which ensures the adaptability of the method to different light intensities and improves the robustness of the prediction model. Figure 8 shows annotation results of the fisheye images under different weather conditions.

To explore the network structure of YOLOX with the best detection effect, YOLOX-s, YOLOX-tiny, and Attention-YOLOX-tiny are tested. The precision (P), recall (R), F_1_ score (F_1_), and average precision (AP) are used to evaluate the target detection model. P is for the prediction result and it is the proportion of correctly predicted positive samples to all predicted samples. R is for the original sample and it is the proportion of correctly predicted positive samples out of all positive samples. F_1_ is an indicator and a trade-off of P and R. AP is the area under the P–R curve. AP can measure the trained model on a single tree prediction. These evaluation indicators are defined as the following formulas:

(12) $$P=\frac{\mathrm{TP}}{\mathrm{TP}+\mathrm{FP}}$$

(13) $$R=\frac{\mathrm{TP}}{\mathrm{TP}+\mathrm{FN}}$$

(14) $$F_{1}\mathrm{score}=\frac{2P\times R}{P+R}$$

(15) $$\mathrm{AP}=\int_{0}^{1}P(R)dR$$

The acquired images were used to make a fisheye image dataset. To increase the training efficiency, Docsmall (an image compression website) was used to compress the images before training. The compressed images were divided into a training set and validation set. The ratio of the training set and validation set was 9:1. The processor used for training was Intel Core I7-10700K, 3.80 GHZ processor, 32 GB memory, 10 GB NVIDIA RTX 3080 GPU. The training parameters are set as shown in Table 2. The evaluation results of each network are shown in Table 3.

**Figure 9:**
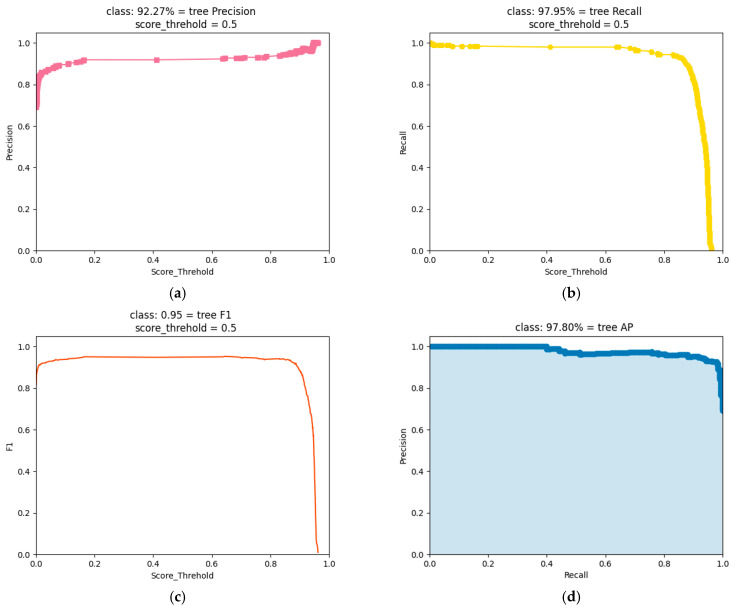
Evaluation results, which include P, R, F_1_, and AP. (**a**) P; (**b**) R; (**c**) F_1_; (**d**) AP.

As shown in Table 3, on the fisheye image dataset of trees, the P of Attention-YOLOX-tiny is 92.27%, R is 97.95%, F_1_ is 0.95, and AP is 97.80%. Most of the performance metrics of Attention-YOLOX-tiny, including R, F_1_, and AP, are better than YOLOX-s and YOLOX-tiny. The evaluation process of the Attention-YOLOX-tiny detection model is shown in Figure 9.

**Figure 10:**
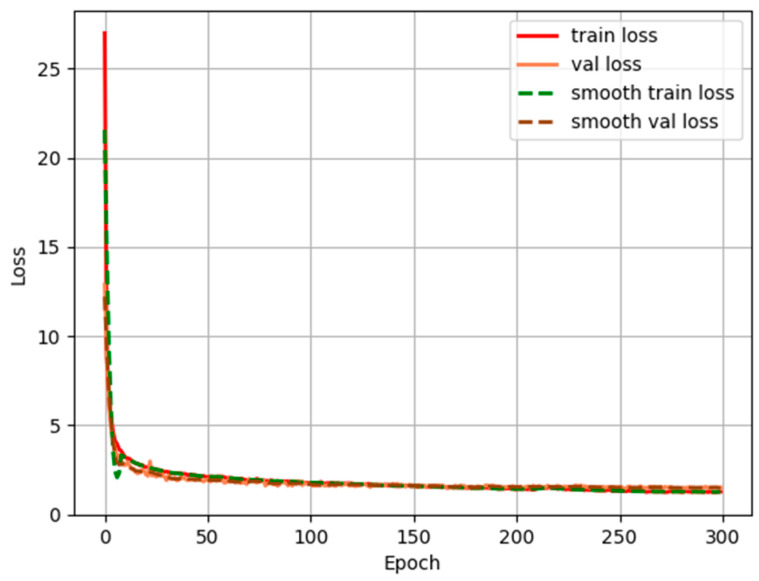
LOSS function change curve.

From the detection indicators in Figure 9, it can be concluded that the prediction results of trees can meet the requirements of accurate detection of trees. The LOSS function curve of the training process is shown in Figure 10.

Figure 10 shows the loss variation curve of Attention-YOLOX-tiny, where the horizontal and vertical axes represent training epochs and loss values. With the increasing number of training iterations, the loss value on the training set, the loss value on the validation set, the smooth loss value on the training set, and the smooth loss value on the validation set of Attention-YOLOX-tiny all decrease rapidly at first, and then gradually decrease. The loss curve of Attention-YOLOX-tiny gradually converges around 2.0 after about 150 iterations. The loss curve has converged which indicates that the predicted output is credible. The trained model has learned the characteristics of the tree under the fisheye distortion and can extract the extreme points.

**Figure 11:**
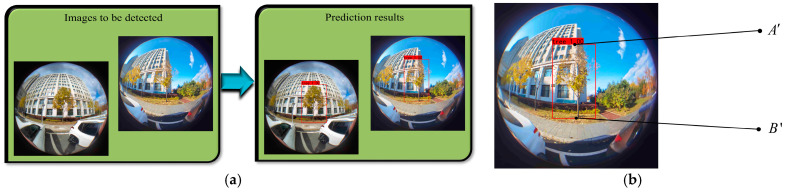
Tree detection and tree extreme point extraction. (**a**) Fisheye image of detection tree; (**b**) Extract extreme points.

Figure 11 shows the detection result of the tree and the extraction result of the extreme points of the tree. Through the above experimental analysis, it can be concluded that Attention-YOLOX-tiny can accurately detect the target object in the picture. By extracting the coordinates of the detection frame in the picture, the coordinates of the extreme point $A^{\prime }\left(u_{A^{\prime }},v_{A^{\prime }}\right)$ and the extreme point $B^{\prime }\left(u_{B^{\prime }},v_{B^{\prime }}\right)$ in the model can be obtained.

### 3.3. Tree Height Calculation

**Figure 12:**
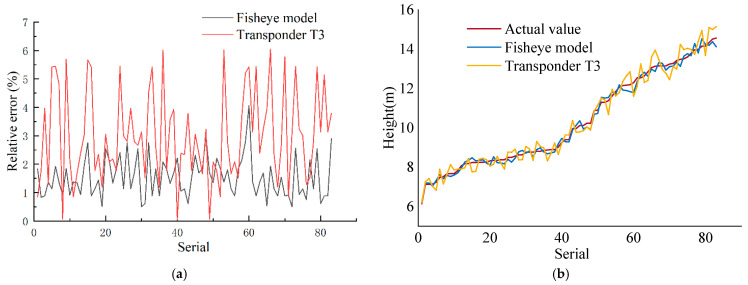
Measurement results. (**a**) Measurement Error; (**b**) Measured value.

The coordinates of $A^{\prime }\left(u_{A^{\prime }},v_{A^{\prime }}\right)$ and $B^{\prime }\left(u_{B^{\prime }},v_{B^{\prime }}\right)$ are the coordinates of the midpoints of the upper and lower frame lines in the image. The fisheye lens measurement model is taken to obtain the predicted tree height. In this experiment, the average value of the ten times measured by the theodolite was taken as the actual value. We selected 83 trees as validation data and for contrastive measurements with Transponder T3. The tree height measurement results are shown in Figure 12; Figure 12a is the comparison of the relative errors of the fisheye model and Transponder T3 and Figure 12b is the comparison of their measurement values.

The experimental results show that the average relative error of the method in this paper is 1.62% and the average relative error of Transponder T3 is 3.23%. Through comparison, it can be found that the average error of this method is significantly smaller than Transponder T3. The calculation result of this method is more stable than Transponder T3.

### 3.4. Wind Interference Experiment

**Figure 13:**
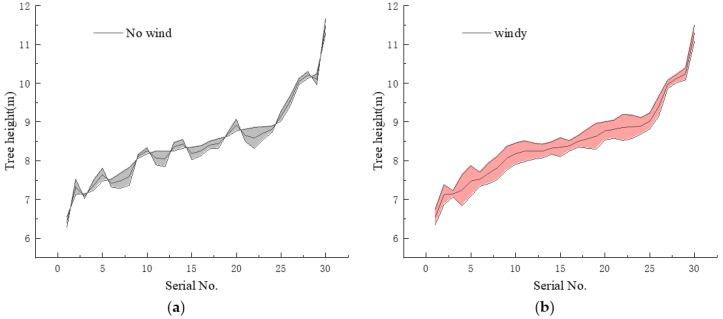
Errors under different measurement conditions. (**a**) The wind is less than level 3; (**b**) The wind is at level 5~6.

The measurement environment in practical applications is variable. To verify the accuracy of this method under windy measurement conditions, a windy day was selected. The wind level was 5~6 (taken from China Weather Network). Transponder T3 does not work correctly in this condition. The fisheye images of 30 trees were obtained and calculated. The experiment shows that under the conditions of wind measurement, the average error of this method is 2.31% and Transponder T3 has completely failed. The calculation result of this method is shown in Figure 13. The shaded part in the Figure 13 is the absolute error. The practicality of this method under the influence of wind is better than that of Transponder T3.

When the wind reaches level 5~6, the measurement effect of the method in this paper is affected because the wind changes the shape of the tree and affects the extreme point coordinates of the tree extracted by Attention-YOLOX-tiny. This eventually leads to an increase in measurement error.

## 4. Conclusions

Compared with the ultrasonic rangefinder to measure tree height, the relative error of the ultrasonic rangefinder was the highest at 6.04%, the lowest was 0.34%, and the aver-age relative error was 3.23%. The highest relative error of the method calculated in this paper is 4.06%, the lowest relative error is 0.5%, and the average relative error is 1.62%. In tree detection, Attention-YOLOX-tiny can accurately and quickly extract the extreme points of trees. Overall, the average relative error of the method in this paper is low, which is better than the ultrasonic rangefinder in measurement accuracy. The method has the advantages of stable measurement, compact structure, and easy portability.

Experiments were carried out to analyze the errors under different measurement conditions. The average relative error of the method in this paper is 2.31% under the condition of level 5–6 wind. Compared with the no-wind condition, the relative error calculated by this method increases slightly under the gale conditions. However, it can still complete the measurement task and maintain good accuracy.

As an important indicator for measuring forest carbon storage, tree height has always been a hotspot in forest research. This study obtains Attention-YOLOX-tiny by improving the target detection network and proposes a new method for measuring tree height based on Attention-YOLOX-tiny. Consisting of a mobile phone and a matching fisheye lens, the measurement device will continue to improve with the rapid development of electronics and manufacturing capabilities. The proposal of more accurate and lightweight detection networks in computer vision can extract the extreme points of trees more quickly and accurately. In future research, tree extremum points can be extracted by faster and more accurate object detection and segmentation networks. The disadvantage of this research is that it is difficult to obtain 3D information about trees only through 2D images; 3D reconstruction of trees through images is the main break-through direction in the future.

## Figures and Tables

**Table 1:** *AB*, *AC*, *AD* calculation results.

Measuring Distance (mm)	Corner Point	Pixel Coordinate	Global Coordinates	Calculated Value (mm)	Measured Value (mm)	Relative Error (%)
320	*A*	(898, 687)	(−285.0330, −375.9156)	656.3162	650	0.9717
	*B*	(810, 2290)	(−300.6751, 280.2142)			
612	*A*	(1091, 966)	(−307.0069, −388.4370)	656.2374	650	0.9896
	*B*	(1062, 2021)	(−310.4134, 267.7916)			
993	*A*	(1179, 1160)	(−375.2495, −395.9568)	657.1972	650	1.1073
	*B*	(1150, 1860)	(−394.4027, 260.9612)			
1502	*A*	(1374, 1282)	(−274.3518, −401.1987)	652.1023	650	0.3234
	*B*	(1358, 1766)	(−292.3145, 250.6562)			
2041	*A*	(1405, 1346)	(−312.3874, −422.4221)	654.4923	650	0.6911
	*B*	(1389, 1707)	(−338.4833, 231.5497)			
320	*A*	(898, 687)	(−285.0330, −375.9156)	647.8777	650	0.3265
	*C*	(2414, 782)	(362.2036, −347.1019)			
612	*A*	(1091, 966)	(−307.0069, −388.4370)	655.2693	650	0.8107
	*C*	(2126, 1000)	(347.9883, −369.4865)			
993	*A*	(1179, 1160)	(−375.2495, −395.9568)	644.3722	650	0.8658
	*C*	(1870, 1195)	(267.0121, −352.6874)			
1502	*A*	(1374, 1282)	(−274.3518, −401.1987)	642.8080	650	1.1065
	*C*	(1850, 1301)	(368.0232, −377.6071)			
2041	*A*	(1405, 1346)	(−312.3874, −422.4221)	641.7176	650	1.2742
	*C*	(1760, 1365)	(328.3721, −387.3684)			
320	*A*	(898, 687)	(−285.0330, −375.9156)	928.6904	919.238	1.0282
	*D*	(2410, 2314)	(344.7874, 306.5740)			
612	*A*	(1091, 966)	(−307.0069, −388.4370)	921.6590	919.238	0.2633
	*D*	(2086, 2065)	(308.7236, 297.3699)			
993	*A*	(1179, 1160)	(−375.2495, −395.9568)	928.6176	919.238	1.0203
	*D*	(1850, 1890)	(244.6171, 281.7389)			
1502	*A*	(1374, 1282)	(−274.3518, −401.1987)	912.5988	919.238	0.7223
	*D*	(1829, 1785)	(335.5699, 277.6474)			
2041	*A*	(1405, 1346)	(−312.3874, −422.4221)	911.4613	919.238	0.8461
	*D*	(1741, 1723)	(291.7372, 260.0696)			
Mean value						0.8231

**Table 2:** Training parameters.

Parameters	Value
Input size	640 × 640
Output size	640 × 640
Learning rate	adaptive
Batch size	8
Epoch	300

**Table 3:** Detection evaluation of different networks.

Model	Epoch	P (%)	R (%)	F_1_	AP (%)
YOLOX-s	300	92.57	95.90	0.94	96.27
YOLOX-tiny	300	93.03	95.90	0.94	97.26
Attention-YOLOX-tiny	300	92.27	97.95	0.95	97.80

## Data Availability

Not applicable.

## Appendix A

**Table A1:** Symbols and abbreviations which appear in the text.

Symbol or Abbreviation	Explanation
*P*	The target point in the world coordinate system.
*P’*	The imaging point corresponding to *P* in the camera coordinate system.
*r’*	The distance from the point *P’* to the optical axis.
*f*	The object square focal length of the optical system.
*w*	The incident angle of the point *P* relative to the optical axis.
*L*	The horizontal distance between the point in the world coordinate system and the center of the fisheye lens.
*λ*	The distortion coefficient.
*θ*	The azimuth of point *P* and the azimuth of point *P’* in the camera coordinate system.
*m_x_* and *m_y_*	The amplification factors.
*k_x_* and *k_y_*	The distortion coefficients of the fisheye image.
*h*	The horizontal distance in the world coordinate system.
*H*	The result was obtained by the measurement system model.
*CBMA*	Convolutional Block Attention Module.
*D_S_*	Source domain.
*T_S_*	Source task.
*D_T_*	Target domain.
*T_T_*	Target task.
*f_T_*	Target function.

**Table A2:** Measuring equipment parameters.

Fisheye Lens			Smartphone		
Attributes	Value	Unit	Attributes	Value	Unit
Thread diameter	17	mm	Size	148.9 × 71.1 × 8.5	mm
Angle	180		Pixel	50	million
Weight	36	g	Weight	175	g
Resolution	4096 × 4096	dpi	Photo resolution	8192 × 6144	dpi
